# Stroke in Takayasu arteritis with concomitant *tuberculosis*: an unusual pediatric case report

**DOI:** 10.1186/s12887-022-03125-4

**Published:** 2022-01-20

**Authors:** Yao Tian, Yu Chen

**Affiliations:** Department of Pediatric Tuberculosis, Shenyang Tenth People’s Hospital, Shenyang Chest Hospital, Shenyang, 110044 Liaoning China

**Keywords:** *Mycobacterium tuberculosis*, Takayasu arteritis, Stroke, Child, Case report

## Abstract

**Background:**

Stroke is a lethal complication of polyarteritis in children. Takayasu arteritis is a rare disease with an unknown etiology and is known to mainly affect young women. In this report, we present the case of a Chinese boy diagnosed with TA results in stroke, originally presenting in the context of latent tuberculosis infection and then developing active tuberculosis.

**Case presentation:**

The patient was a 14-year-old child who developed a latent tuberculosis infection at age 5 after coming in close contact with his grandfather, who had tuberculosis. However, he did not receive any anti-tuberculosis medications at that time. At age 9, he was hospitalized for symptoms of "dizziness and headache" and was diagnosed with Takayasu arteritis and hypertension; however, tuberculosis was not diagnosed. Only antihypertensive drugs were administered without considering the possible pathogenic factors of tuberculosis infection. At age 14, he was rehospitalized for "fever and cough" and was diagnosed with active pulmonary tuberculosis as an analysis of his fiberoptic bronchoscopy sample using the Gene-Xpert assay was positive for *Mycobacterium tuberculosis*. However, after 2 months of taking oral anti-tuberculosis drugs, his blood pressure continued to rise, and he presented with numbness and weakness of the right limb and a deviation of the right side of his mouth. Computed tomography angiography of his head and neck revealed that the walls of the left subclavian artery and bilateral vertebral arteries were thickened, and the lumen was significantly narrowed. In a recent examination, magnetic resonance imaging and diffusion-weighted imaging of the head showed infarctions in the right basal ganglia area close to the left lateral ventricle. Our patient was treated with methotrexate, tocilizumab and glucocorticoids to control he continued active vasculitis.

**Conclusions:**

The possible association of tuberculosis and Takayasu arteritis complicated by stroke needs to be considered, especially in children who had prior contact with a family member with tuberculosis infection. The temporal relationship between TA and infection with Mycobacterium tuberculosis in our patient suggests a compelling link that demands further investigation.

## Background

Takayasu arteritis (TA) is a granulomatous vasculitis of large vessels that affects the aorta and its major branches. The ascending and descending aorta and the subclavian, carotid, and vertebral arteries are the most commonly involved blood vessels. TA mainly affects young women, and its incidence is 2.6 per million per year [1]. A cohort study indicated that 77% had disease onset between the ages of 10 and 20 years, with a time from onset of symptoms to diagnosis of two to 11 years in 78% [2]. The pathophysiology of TA may be related to cell-mediated immune disorders and genetic susceptibility. Therefore, TA is mostly considered to be related to genetics, endocrine abnormalities, infections (*Streptococcus*, *Mycobacterium tuberculosis (M. tuberculosis)*, viruses, etc.). An estimated 67 million children worldwide have tuberculosis infection (latent tuberculosis infection, LTBI) and are at risk of developing active tuberculosis [3]. *An association between TA and infection with M. tuberculosis has been suggested but not proven.* LTBI and active tuberculosis have been observed in 20%–82% and 6.3%–20% of TA cases, respectively [4]. The most common signs of TA include hypertension, increased difference between diastolic and systolic blood pressure, dizziness, and headache. Cerebrovascular accidents as complications of TA may be fatal. Therefore, timely diagnosis and effective treatment strategies are of great importance. This report describes a rare case of a boy with stroke in TA with concomitant tuberculosis. Ethical approval for this report was obtained from the ethics committee of our hospital.

## Case presentation

The patient was a 14-year-old Chinese boy who had a positive interferon-gamma release assay (IGRA) result at the age of 5 years after being in close contact with his grandfather, who had active tuberculosis. At the age of 9 years, he was admitted to a large-scale pediatric hospital for "dizziness and headache" and was diagnosed with severe hypertension caused by polyarteritis involving the renal arteries. He was treated with antihypertensive drugs but was not administered any antituberculosis drugs for *M. tuberculosis* infection at that time. It should be noted that the patient had received the Bacillus Calmette-Guerin vaccine as a child in China.

The patient initially complained of fever, cough, shortness of breath, fatigue, and weight loss for 1.5 months before presentation, with subsequent development of pain on the right side of his chest. On presentation, he was admitted for shortness of breath after failed outpatient antibiotic treatment for presumed pneumonia.

Upon admission (March 2019), the patient presented with fever, cough, shortness of breath, and chest pain on the right side, with a respiratory rate of 18 breaths/min and a heart rate of 100 beats/min. His blood pressure was 216/112 mmHg, and subsequent measurements revealed a blood pressure gradient between the upper limbs, with 170/80 mmHg recorded on the left arm and 220/120 mmHg on the right arm.

**Fig. 1:**
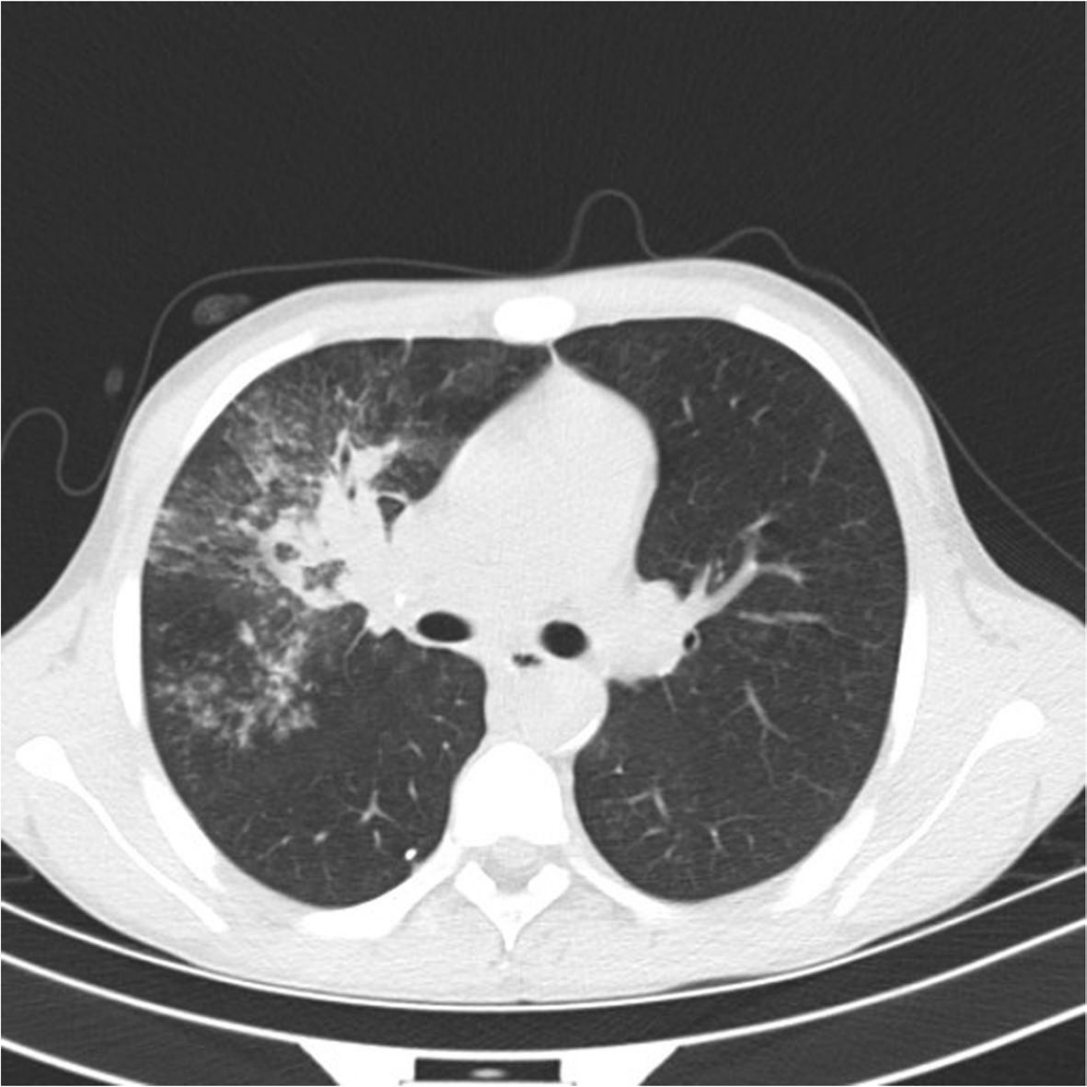
The chest CT image indicates uneven high-density shadows of patchy, stippled, stripe, and nodular shapes in the upper and lower lobes of the right lung, thickened adhesions in the adjacent pleura, and enlarged and calcified mediastinal lymph nodes

Cardiopulmonary examination revealed rough breathing sounds in both lungs and vascular murmurs in the left part of the neck and left supraclavicular fossa. Chest computed tomography indicated uneven high-density shadows of patchy, stippled, stripe, and nodular shapes in the upper and lower lobes of the right lung, thickened adhesions in the adjacent pleura, and enlarged and calcified mediastinal lymph nodes (Fig. 1). Fiberoptic bronchoscopy revealed congestion and edema of the bronchial mucosa in the upper lobe of the right lung, and the surface of the B3 subsegment bronchial mucosa was covered with a cheese-like substance. A T-SPOT. TB test returned positive results again. His bronchoscope alveolar lavage fluid sample tested positive in the Xpert MTB/RIF test (Cepheid, USA), and rifampin resistance was not detected. The *M. tuberculosis* complex was discovered in bronchoscopic alveolar lavage fluid by high-throughput sequencing.

Induced sputum was sent for acid-fast bacillus smear three times, and all the results were negative. Sputum for MGIT 960 culture (BD, USA)was positive. A drug sensitivity test (DST) was performed and found to be sensitive to isoniazid, rifampicin ethambutol and streptomycin. The remainder of an extensive infectious workup showed negative results. Therefore, the patient was placed on a four-drug anti-tuberculosis regimen that included rifampin, isoniazid, pyrazinamide, and ethambutol for the treatment of confirmed active tuberculosis. Laboratory examinations on admission showed that he had a white blood cell count of 5.5 × 10^9^/L (normal range, 3.5–9.5 × 10^9^/L) with 54.2% neutrophils, hemoglobin level of 126 g/L (normal range, 130–175 g/L), platelet count of 286 × 10^9^/L (normal range, 125–350 × 109/L), hypersensitive C-reactive protein level of 31.6 mg/L (normal range, 0–4 mg/L), procalcitonin level of 0.077 ng/mL (normal range, 0.02–0.05 ng/mL), and PaO_2,_ 81 mmHg (80–100 mmHg). Biochemical examination showed that his alanine aminotransferase level was 10 U/L (normal range, 9–50 U/L), and his aspartate aminotransferase level was 16 U/L (normal range, 15–40 U/L). His erythrocyte sedimentation rate was 28 mm/h (normal range, 0–15 mm/h). Cerebrospinal fluid findings showed leucocyte count of 3 × 10^6^/L (normal range, 0–8 × 10^6^/L), lymphocyte of 100%, protein of 0.26 g/L (normal range, 0.15–0.45 g/L), glucose concentration of 3.05 mmol/L (normal range,2.5–4.5 mmol/L), chloride of 121 mmol/L (normal range, 120–130 mmol/L).

**Fig. 2:**
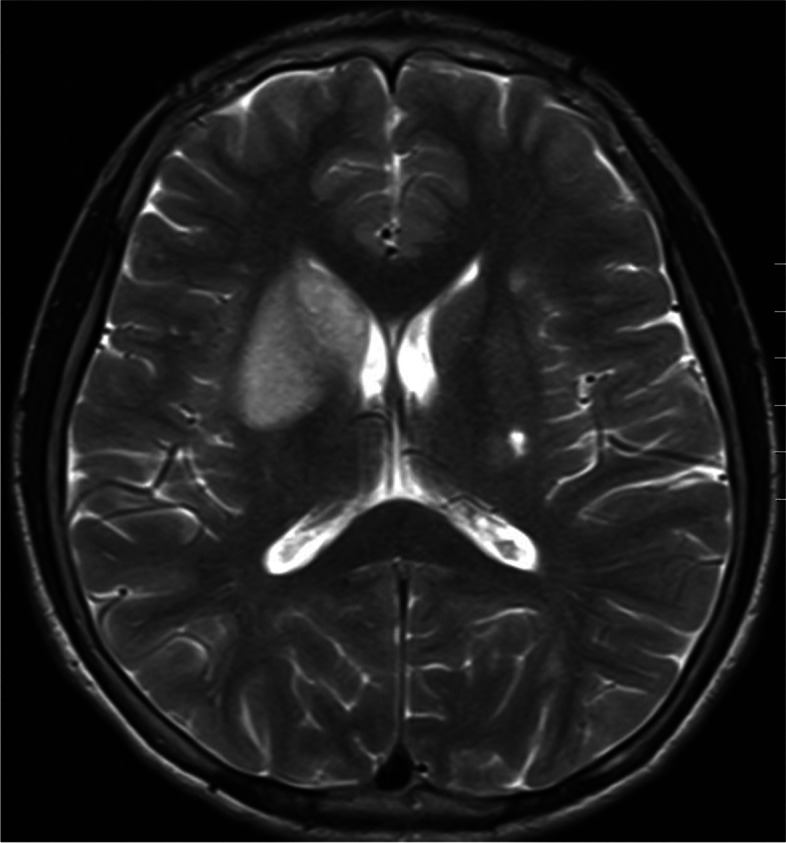
MRI shows patchy, spot-like, long, T2 signal shadows in the right basal ganglia and around the left lateral ventricle

**Fig. 3:**
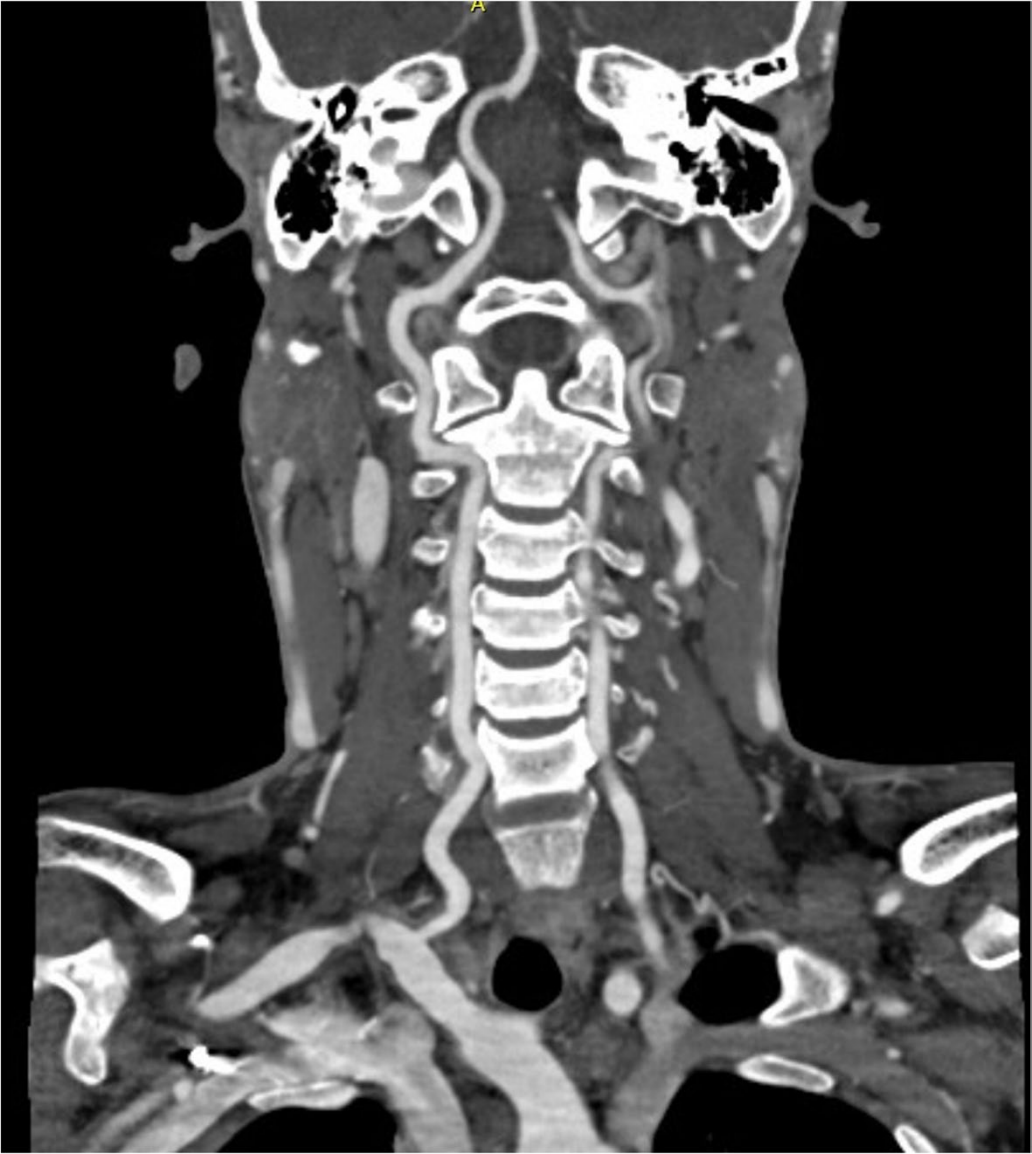
The CT angiography image indicates localized thickening of the vascular wall at the initial segment of the left vertebral artery with moderate-to-severe stenosis of the lumen and mild stenosis of the lumen at the initial segment of the right vertebral artery

**Fig. 4:**
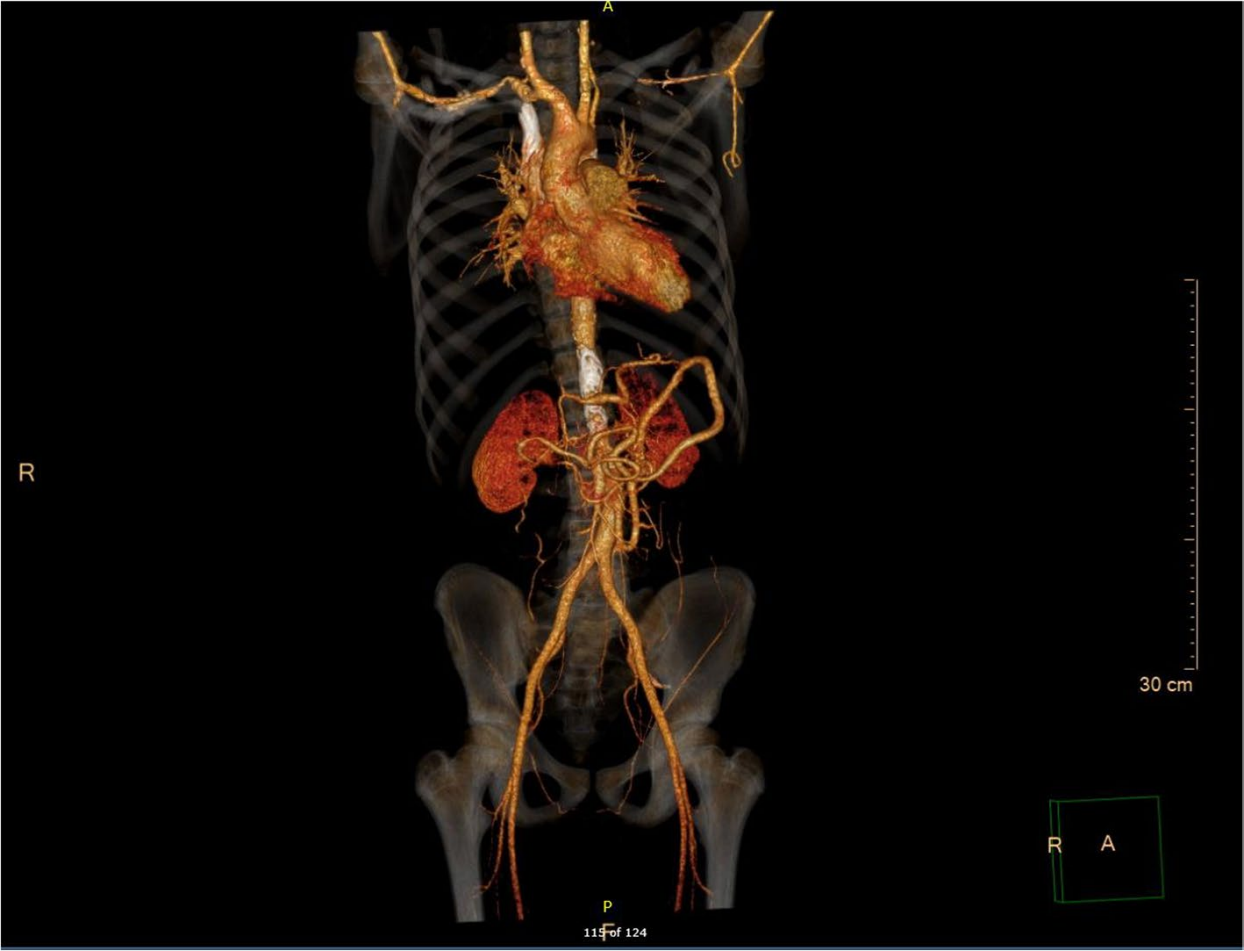
The lumen at the initial segment of the left subclavian artery is slightly narrowed, the vascular walls at the proximal and middle segments are diffusely thickened with local lumen occlusion and moderate stenosis, and the distal lumen is slender. The wall of the thoracic-abdominal aorta is slightly thickened with multiple calcifications, whereas the lumen is slightly narrowed. Obvious luminal stenosis can be observed at the beginning of the abdominal trunk, as well as at the beginning portion of the bilateral renal arteries

Two months after discharge, the child's fever, cough, chest pain, and shortness of breath were alleviated, and this was reflected in radiographic images of his lungs. However, he began experiencing numbness and weakness of the right limbs, and his mouth was askew on the right side; his blood pressure also increased up to 220/120 mmHg. Magnetic resonance imaging (MRI) and diffusion-weighted imaging (DWI) of the head showed patchy, spot-like, long, T2 signal shadows in the right basal ganglia and around the left lateral ventricle (Fig. 2). A diagnosis of recent cerebral infarction was then rendered. During hospitalization, he was administered cattle encephalon glycoside and ignotin injection for nourishing the cranial nerves; urapidil, nifedipine, and terazosin for lowering blood pressure; and aspirin for antiplatelet effects. Computed tomography angiography (CTA) of the head, neck, chest, and abdomen was performed to evaluate his vasculitis. CTA showed active disease, localized thickening of the vascular wall at the initial segment of the left vertebral artery with moderate-to-severe stenosis of the lumen and mild stenosis of the lumen at the initial segment of the right vertebral artery and the branches of the middle cerebral artery (Fig. 3). The lumen at the initial segment of the left subclavian artery was slightly narrowed, the vascular walls at the proximal and middle segments were diffusely thickened with local lumen occlusion and moderate stenosis, and the distal lumen was slender. The wall of the thoracic-abdominal aorta was slightly thickened and had multiple calcifications, whereas the lumen was slightly narrowed. Luminal stenosis became apparent at the beginning of the abdominal trunk, and the initial portion of the bilateral renal arteries was also obviously narrowed (Fig. 4). Considering the apparent high activity and extensive vasculitis changes that may be caused by immune damage in the patient, glucocorticoid, methotrexate, and tocilizumab injections were used to control severe disease.

After 3 months of standard treatments, the patient’s clinical symptoms improved to some extent; however, he was still unable to move his right lower limb at liberty. Laboratory examinations showed an erythrocyte sedimentation rate of 10 mm/h, a blood pressure of 150/85 mmHg, and mild anemia (hemoglobin level of 115 g/L, normal range: 130–175 g/L) but no other abnormalities. The patient completed 9 months of anti-tuberculosis treatment in December 2019. His clinical symptoms and lung findings improved considerably, and sputum for acid-fast bacillus smear and MGIT 960 culture was negative. After 1 year of treatment for TA, the administration of methotrexate and tocilizumab injection was discontinued, and the oral dose of the glucocorticoid was gradually reduced to 5 mg. The patient was still undergoing antihypertensive therapy at the time this report was written. He is being managed by different rheumatologists as his condition stabilizes.

## Discussion and conclusions

TA is a vasculitis of large blood vessels that mainly affects the aorta and its major branches, such as the subclavian artery and carotid artery. The stenosis and occlusion of the carotid and vertebral arteries occurring in TA can cause brain ischemia to varying degrees. Stroke caused by ischemia is a potential complication in TA. A previous study revealed that 3.9% of 79 patients with TA appeared to have stroke secondary to large-vessel occlusion [5]. A diagnosis of recent cerebral infarction was rendered in the present case by head MRI + DWI. Hence, TA should be considered as a differential diagnosis in children with stroke.

Although the etiology of TA is not fully understood, it is speculated that it may be related to heredity, endocrine abnormalities, and infection factors. The prevalence of LTBI in patients with TA is 20–82% [4]. Although most of the studies show a high prevalence of TB, it is not possible to establish a causal relationship. There have been a few reports of pediatric cases in which TA with concomitant tuberculosis led to rare complications such as ocular tuberculosis and skin tuberculosis and even fatal complications such as myocardial infarction and dissecting aneurysm.[6–9]. To the best of our knowledge, this is the first report of a pediatric case of TA with concurrent stroke associated with *M. tuberculosis* infection. In countries with a high tuberculosis burden, most LTBIs in children are associated with close contact with family members who have active tuberculosis. The World Health Organization proposed that regardless of the epidemiological background of tuberculosis, LTBI screening and preventive treatment are beneficial for infants and children under 5 years old who have had close contact with patients with pulmonary tuberculosis in all settings [10]. However, according to statistics collated in 2018 and 2019, 782,952 children under the age of 5 who had close contact with family members with tuberculosis received preventive treatments for tuberculosis, accounting for only 20% of the 5-year target number (4 million) [11]. Although there is no conclusive evidence to establish an acausal relationship, an apparent association between TA and *M. tuberculosis* infection has been oted previously. There are currently two assumptions regarding the relationship between LTBI and TA. The first hypothesis is that loss of tolerance against self-stress proteins is a primal pathogenic event in TA, whereas the extensive sequence homology between mycobacterial and human stress proteins leads to epiphenomenal cross-reactions [12]. The second hypothesis is based on the possibility that arteritis results directly from LTBI [13]. Our case suggests the temporal relationship between TA and infection with Mycobacterium tuberculosis. Unfortunately, after being in close contact with a family member with active tuberculosis at age 5, the child was only screened for LTBI but was not administered preventive treatment. We suggest that greater care should be taken with latent TB screening in TA patients, although this potential relationship is not yet understood.

TA can trigger brain ischemia to varying degrees through the involvement of the carotid and vertebral arteries, manifesting as dizziness, headache, convulsions, hemiplegia, etc., and can induce severe renal arterial hypertension by involving the renal artery. Generalized TA has all the characteristics mentioned above and is also characterized by extensive involvement of blood vessels and diverse lesions. Most patients have severe disease and develop serious complications, such as cerebrovascular accidents, heart failure, aneurysm rupture, and myocardial infarction, all of which can lead to death. In the present case, in addition to persistent renal hypertension, the patient had a stroke secondary to TA with concomitant tuberculosis. Anti-tuberculosis therapy, anti-platelet therapy and anti-hypertensive drugs were administered during the initial active phase, and glucocorticoids, methotrexate, and tocilizumab injection were used to control severe disease. The effect of anti-tubercular drugs on TA symptoms remains debatable among authors [14]. It is also unclear whether anti-tuberculosis therapy can prevent TA progression and serious complications. However, it is essential to note that most studies have combined corticosteroid and antitubercular drugs to treat the cooccurrence of TA and active TB.

Abnormalities in angiographic examinations are considered the gold standard for the diagnosis of TA in children. The results of routine angiography or arterial CTA and magnetic resonance angiography suggest that dilation, aneurysm, stenosis, occlusion, and thickening of the arterial wall occurred in the aorta and its main branches or pulmonary artery [15]. Concurrent stroke, which is more commonly noted in the basal ganglia, brainstem, cerebellum, brain lobe, and cerebral watershed, can be detected using head computed tomography or MRI [16]. Glucocorticoids and traditional disease-modifying anti-rheumatic immunosuppressants are currently effective drugs for TA. Studies have shown that biological agents such as tocilizumab effectively treat TA and reduce recurrence with few adverse reactions [17]. Hypertension caused by renal artery stenosis can be managed with urapidil, nifedipine, and terazosin, whereas stroke can be treated with antiplatelet drugs, such as aspirin. The clinical assessment of TA activity is challenging because some clinical symptoms, such as low fever, night sweats, fatigue, weight loss, and persistently elevated inflammatory markers, also appear during tuberculosis activities. Hypertension caused by renal artery stenosis can be aggravated owing to the side effects of hormone-induced water-sodium retention. In addition, methotrexate, tocilizumab and glucocorticoids are preferred due to disease progression. As tocilizumab is used, the risk that latent tuberculosis infection progresses to active *M. tuberculosis* infection should be considered, especially in tuberculosis-endemic countries [18]. In the present case, the child was considered to have TA at the age of 9. However, no standardized follow-up was planned or implemented; therefore, the treatment of active TA was delayed.

Children under 5 years old who have had close contact with a family member with tuberculosis and children with positive IGRA results belong to the high-risk group that requires interventional treatment. Ninety to 95% of these *M. tuberculosis*-infected individuals are in a latent asymptomatic infection state for a long time, but 5–10% of them will progress to active TB during their lifetime. Diagnosing paediatric TB are challenging. Thus, the diagnostic algorithm relies on signs, symptoms, evidence of *M. tuberculosis* infection (a positive IGRA), history of contact with active TB disease and the results of chest X-ray, liquid culture and molecular tests (Xpert MTB/RIF). Fiberoptic bronchoscopy is helpful for detecting bronchial tuberculosis and obtaining bacterial specimens. Acquiring qualified samples for molecular biology or high-throughput sequencing tests is important for rapid diagnosis. The sensitivity of Xpert MTB/RIF for the pathogenic diagnosis and clinical diagnosis of tuberculosis using the bronchoalveolar lavage fluid of children is 88% and 33%, respectively [19]. In pathogenic diagnosis, high-throughput sequencing technology can identify infectious diseases early, quickly, and accurately [20].

Greater attention needs to be paid to TA-induced severe complications of stroke, especially in the young. Anti-tuberculosis therapy, methotrexate, tocilizumab and glucocorticoids can be used to control active tuberculosis and TA. The temporal relationship between TA and latent or active tuberculous infection in our patient suggests a potential association that demands further investigation.

## Data Availability

All data and material collected during this study are available from the corresponding author upon reasonable request.

## Abbreviations

TA: Takayasu arteritis
LTBI: Latent tuberculosis infection
IGRA: Interferon-gamma release assay
CTA: Computed tomography angiography
MRI: Magnetic resonance imaging
*M. tuberculosis*: *Mycobacterium tuberculosis*
